# Microfluidic Separation of Blood Cells Based on the Negative Dielectrophoresis Operated by Three Dimensional Microband Electrodes

**DOI:** 10.3390/mi11090833

**Published:** 2020-08-31

**Authors:** Tomoyuki Yasukawa, Junko Yamada, Hitoshi Shiku, Tomokazu Matsue, Masato Suzuki

**Affiliations:** 1 Graduate School of Material Science, University of Hyogo, 3-2-1 Kouto, Kamigori, Ako, Hyogo 678-1297, Japan; suzuki@sci.u-hyogo.ac.jp; 2 Graduate School of Environmental Studies, Tohoku University, 6-6-11, Aramaki, Aoba, Sendai 980-8579, Japan; j.kinop@gmail.com (J.Y.); matsue@tohoku.ac.jp (T.M.); 3 Graduate School of Engineering, Tohoku University, Sendai 980-8579, Japan; hitoshi.shiku.c3@tohoku.ac.jp

**Keywords:** cell separation, dielectrophoresis, fluidic flow, label-free, slanted electrodes

## Abstract

A microfluidic device is presented for the continuous separation of red blood cells (RBCs) and white blood cells (WBCs) in a label-free manner based on negative dielectrophoresis (n-DEP). An alteration of the electric field, generated by pairs of slanted electrodes (separators) that is fabricated by covering parts of single slanted electrodes with an insulating layer is used to separate cells by their sizes. The repulsive force of n-DEP formed by slanted electrodes prepared on both the top and bottom substrates led to the deflection of the cell flow in lateral directions. The presence of gaps covered with an insulating layer for the electric field on the electrodes allows the passing of RBCs through gaps, while relatively large WBCs (cultured cultured human acute monocytic leukemia cell line (THP-1 cells)) flowed along the slanted separator without passing through the gaps and arrived at an edge in the channel. The passage efficiency for RBCs through the gaps and the arrival efficiency for THP-1 cells to the upper edge in the channel were estimated and found to be 91% and 93%, respectively.

## 1. Introduction

Dielectrophoresis (DEP) has been used to detect bacteria [1], measure the electric properties of cells [2,3], and to form cell patterns [4,5,6,7,8] to apply to biomedical sciences [9]. Additionally, it has been especially studied as an external force for separating cells in microfluidic devices [10,11,12]. When the external force with the different direction of fluidic flow is applied to cells introduced in a fluidic channel, the flow position of the responsible cells to its external force will be gradually shifted in the channel, resulting in the separation from the non-responsible cells. In the early stage of the study of DEP separation of cells, an upward vertical force by a repulsive force of negative DEP (n-DEP) has been employed to develop field-flow fractionation based on the DEP force (DEP-FFF) [13,14,15,16,17]. The cells that received n-DEP from the bottom of the channel rapidly flowed to downstream compared to the others, due to the levitation of cells to the center region in the vertical direction of the channel. However, the introduction of cell suspensions as a plug flow makes the continuous separation difficult in DEP-FFF. Microfluidic devices with electrode arrays arranged on both side walls have also been employed to apply n-DEP forces to cells in the lateral direction [18]. However, the difficulty of the arrangement of thick electrodes with the height of several tens of micrometer and the sealing without a leak of solution has prevented the fabrication of devices.

Slanted electrodes have been applied to achieve the lateral displacement of cells in the fluidic channel [19]. In the devices, band electrode arrays were slantingly placed to the flow direction in the channel. When cells transported parallelly in the channel by the hydrodynamic force reached at the slanted electrodes, cells balanced between hydrodynamic force and n-DEP force, resulting in the cell flow was deflected along the slanted electrodes. On the other hand, when the n-DEP forces for the cells are smaller than the hydrodynamic force, cells will penetrate across the slanted electrodes. Thus, the different types of cells with different electric properties are separated at the slanted electrodes and eluted through different outlets arranged at different positions in the lateral direction. Devices that adapted the slanted electrodes were used for the particle sorting [20], the separation of platelets [21] and cancer cells [22] from bloods, and the separation of differentiated products from stem cells [23]. However, the electrode arrays were placed on the bottom substrate in these works. In this configuration, the electric fields generated in the channel drastically decreases with the increasing of the height from the electrodes on the bottom substrate. The levitation of cells by n-DEP will decrease the separation effect. Recently, the electrode geometries of slanted electrodes were investigated by the simulation of the induced electric field for the effective and high throughput separation of particles [24].

In contrast, three-dimensional (3D) slanted electrodes with same design on both top and bottom substrates were also studied to separate cells [25]. Cells experienced a stronger n-DEP force at the 3D electrode pairs compared to planar electrode arrays on the bottom substrate. The 3D slanted electrodes were used to separate particles with their size [26] and bacteria expressed by specific surface markers from subpopulations [27,28]. Previously, we applied the 3D slanted electrodes to accumulate particles at the center of channels for developing the rapid immunosensing [29,30]. In general, the use of straight electrodes permits the separation to fragments of cells transported to the lateral direction by n-DEP and of cells penetrated across electrodes. The slanted electrodes with different length and angle were developed to separate the subpopulation multiples [31,32]. We also fabricated the electrode array with different gaps in the channel and applied it to control the flow position of each cell with different sizes [33,34].

This paper studies a continuous separation of red blood cells (RBCs) and white blood cells (WBCs) in a label-free manner based on the n-DEP force formed by the 3D slanted electrodes with the gaps covered with insulating layer on pairs of electrodes where the electric field is relatively weak compared to other regions on the electrodes. The complete blood count (CBC) is a common blood test to measure total numbers of RBCs, WBCs and platelets, with abnormal values of CBCs used to screen diseases. It is useful to develop the simple and non-inversive technique for separating WBCs from RBCs, as there are more than 1000 times as many RBCs as WBCs in blood.

## 2. Materials and Methods

### 2.1. Fabrication of the Fluidic Device for Dielectrophoresis (DEP) Separation

**Figure 1:**
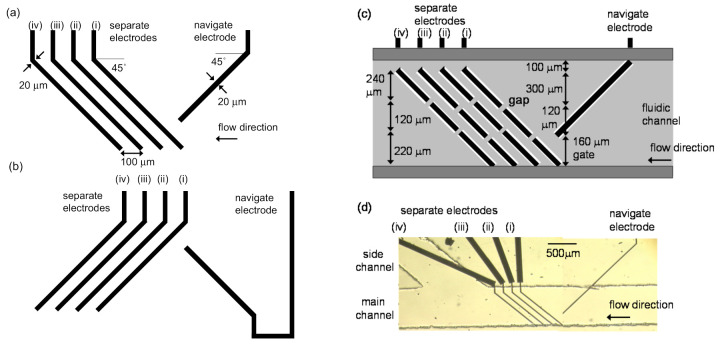
Designs of (**a**) bottom and (**b**) top substrates with Pt electrodes for navigator and separator electrodes; (**c**) bottom substrate covered with the insulating layer (light gray) and attached double-sided adhesive films (dark gray); (**d**) image of the bottom substrate with film for the main and side channels. There are four electrodes (i), (ii), (iii) and (iv) for the separator.

The electrode pattern was fabricated on a glass slide by the conventional photolithography and Ti and Pt sputtering. Figure 1a,b shows the designs of the bottom and top substrates with Pt electrodes, respectively. The navigate electrode with 20 μm wide was set 45° against the flow direction. The angle of four separate electrodes with 20 μm wide were oppositely arranged at 45°. Each separate electrode was arranged with 100 μm gaps. The length of the navigate electrode and separate electrodes were set at 600 μm and 820 μm, respectively. The substrates were covered with negative photoresist (SU-8 3002, MicroChem Corp., Newton, MA, USA) except the electrodes. Figure 1c shows the bottom substrate covered with the insulating layer which was colored by the light gray. Importantly, two gaps that were regions covered with the insulating layer (20 × 20 μm), were provided on each separate electrode. Double-sided adhesive films (regions colored by the dark gray in Figure 1c) with 50 μm thick (Nitto Denko Co., Osaka, Japan) was arranged on the bottom substrate to define the fluidic channel. The insulating layer with same design for the bottom substrate was also prepared on the top substrate. Then, the top substrate was mounted on the bottom substrate with the correspondence to each electrode on both top and bottom substrates. Two gaps on separate electrodes (i) and (iii) were provided on regions of 160 and 280 μm from the edge of the lower side of fluidic channel (Figure 1c). Two gaps for separate electrodes (ii) and (iv) were also provided on regions of 220 and 340 μm. Figure 1d shows the image of the bottom substrate with film for the main and side channels with 680 μm width. An inlet was prepared on the right end of the main channel, and two outlets were also prepared on the left end of the main channel and the end of the side channel by drilling on the upper substrate. Cells flowed from the right side of the main channel. Electric fields for the DEP force were formed between electrodes on the upper and bottom substrates, because the navigate and separate electrodes were directly exposed to the solution due to the removal of photoresist.

### 2.2. Preparation of Mouse Erythrocytes and Human Acute Monocytic Leukemia Cell Line

Mouse erythrocytes (RBCs) were obtained by collecting blood directly from heart of female mice of 30 ages of the weeks. Human acute monocytic leukemia cell line (THP-1 cells) was obtained from the Cell Resource Center for Biomedical Research, Institute of Development, Aging and Cancer Tohoku University. THP-1 cells were grown in RPMI 1640 medium (Invitrogen Japan KK, Tokyo, Japan), containing 10% fetal bovine serum (Invitrogen), 50 units mL^−1^ penicillin (Invitrogen), and 50 μg mL^−1^ streptomycin (Invitrogen), at 37 °C under 5% CO_2_. Suspensions of RBCs (1.0 × 10^8^ cells mL^−1^) and THP-1 cells (2.0 × 10^6^ cells mL^−1^) were respectively prepared in DEP medium consisting of 200 mM sucrose and adjusted the conductivity to 51 mS m^−1^ with a phosphate buffered saline (PBS, 137 mmol L^−1^ NaCl, 2.7 mmol L^−1^ KCl, 8.1 mmol L^−1^ Na_2_HPO_4_, 0.15 mmol L^−1^ KH_2_PO_4_). The average diameters of RBCs and THP-1 were estimated and found to be 6.7 ± 0.7 μm and 16.4 ± 0.5 μm, respectively. We can easily distinguish the cell types by the images, because the diameter of THP-1 cells is more than twice as large as that of RBCs. The shape of RBCs maintained oval biconcave disk in DEP medium used in the present work. The cell suspension was introduced into the device from the inlet to separate cells with their sizes.

### 2.3. Regulation of Cell Position in The Fluid Channel

The channel was treated with the water containing blocking reagent N-101 (NOF Co, Tokyo, Japan) by flowing at 0.1 mm s^−1^ for 30 min to eliminate non-specific binding of cells. Mixtures of RBCs (1.0 × 10^8^ cells mL^−1^) and THP-1 cells (2.0 × 10^6^ cells mL^−1^) were prepared in the DEP medium and introduced into the channel in the device at 150 μm s^−1^ by using the pressure driving pump. AC voltage of 30 V_pp_ in the n-DEP frequency region (25 kHz) was applied to the pair of the navigator electrodes on the top and bottom substrates. AC voltage (25 kHz) with the intensity of 10–16 V_pp_ was applied to each set of separator electrodes. Cells flowing in the channel were observed by an optical microscope (Diaphot300, Nikon Co., Tokyo, Japan) equipped with a digital camera (Coolpix Nikon Co., Tokyo, Japan) and a video monitor (Apple Studio Display, Apple Inc., Cupertino, CA, USA). Sinusoidal voltage was applied using a multichannel function generator (wave factory WF1945, NF Co., Yokohama, Japan). After the voltage was applied, we investigated the passage efficiency for RBCs and arrival efficiency for THP-1 cells, which were defined as a number of RBCs through the gaps on the separate electrodes and a number of THP-1 cells arrived at the upper edge of main channel (Figure 1c) compared to related cells through the gate between the top of the navigate electrodes and separate electrodes (i), respectively.

## 3. Results

### 3.1. DEP Behavior of Red Blood Cells (RBCs) by Using Castellated Electrode

The dielectrophoretic behavior of RBCs suspended in the DEP medium (51 mS m*^−^*^1^) were investigated by using the castellated electrode fabricated by indium-tin oxide (ITO) substrate. When AC voltage with the frequency of 25 kHz was applied to bands of the castellated electrode, RBCs were accumulated on the regions between the projecting parts in the single bands to form triangular shape (Figure S1a). Relatively weak electric fields were formed at their regions by applying AC voltage. Thus, RBCs experienced n-DEP that is the repulsive force against the strong electric field regions. On contrast, RBCs were accumulated on the regions between the projecting parts of neighbor bands with relatively strong electric fields to form rectangle shape by applying AC voltage with the frequency of 1.0 MHz (Figure S1b). This is due to positive DEP (p-DEP) that is the attractive force to the strong electric field regions. The frequency dependence of the direction of cell manipulation was investigated by applying AC voltage with different frequencies. RBCs experienced n-DEP by applying AC voltage under 100 kHz, while RBCs experienced p-DEP over 400 kHz. The cross-over frequency, where the net dielectrophoretic force becomes zero, could be found between 100–400 kHz. The DEP behavior of THP-1 cells has been investigated in previous our report [34]. Similar properties were observed for THP-1 cells with the lower shift of the cross-over frequencies. Both cells experience n-DEP in the frequency region below 50 kHz in this conductivity of the solution.

### 3.2. Numerical Calculation of Electric Field for the Separator

**Figure 2:**
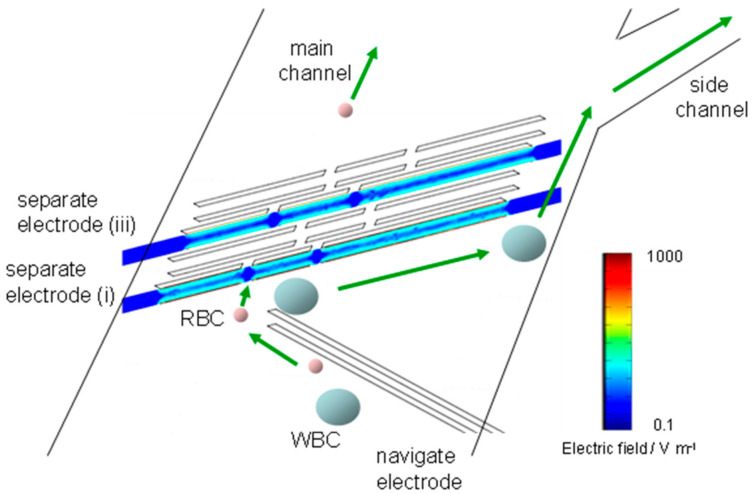
Cross-sectional plots of electric fields along centers of the separation electrodes (i) and (iii).

The distribution of the electric field strength for the separator in the device was calculated by the finite element method (FEM) solver (COMSOL MultiphysicsTM, Stockholm, Sweden). Figure 2 shows cross-sectional plots of electric fields along centers of the separation electrodes (i) and (iii) when a potential difference (14 V) is applied to the pairs of the top and bottom electrodes for the separation electrodes (i) and (iii). The navigation and separation electrodes are represented by the white rectangles in Figure 2. The distance between the pair of electrodes was set at 55 μm. Both cells direct to the edge without side channel along the navigation electrodes by the repulsive force of n-DEP generated by the navigation electrodes, resulting in the accumulation at the edge (left edge in Figure 2). The both cells reached at the separator (i) are again repelled and deflected to another edge of the channel with same manner. Relatively strong electric filed is generated between the separation electrodes, while relatively low electric field region appeared in the regions at the gaps colored with blue in Figure 2. When hydrodynamic force for the cells is larger than repulsive force generated by n-DEP at the gaps, the cells will flow downstream through gaps. On the other hand, when the cells experience the strong repulsive force at the gaps compared to the hydrodynamic force, the cells will be brought toward the edge with the side channel along the separator.

We studied forces acting on RBCs and THP-1 cells at the separation electrodes by the theoretical calculation. Cells repelled from the electric field generated by the separation electrode arranged on the top and bottom substrates. The repulsive force by n-DEP increases with the decreasing distance between cells and the separation electrodes. When the repulsive force balances with the vertical component of hydrodynamic force to the separator, cells move along the separation electrodes. The maximum DEP force, *F_DEP_*(N), which the particles experience half-way between the electrodes, has been calculated [35]:

(1) $$F_{DEP}=\frac{27}{32}\pi^{2}\epsilon_{m}{\left(\frac{a}{h}\right)}^{3}Re\left(K\left(\omega \right)\right)V_{RMS}^{2}$$

where *ε*_m_ is the permittivity of the suspending medium (F/m), a the cell radius (m), h the distance between electrodes (m), *Re*(*K*(*ω*)) the real part of the Clausius–Mossotti factor, and V_RMS_ the root mean square voltage applied (V). If we employ nominal value for the permittivity of the suspending medium, *ε*_s_ = 80ε_0_ (F/m), and maximum *Re*(*K*(*ω*)) value for n-DEP, −0.5, calculated *F*_DEP_ for RBCs was approximately 10 pN by applying the voltage 14 Vpp between the separation electrodes on the top and bottom substrates. In a constant flow, the particles experience a hydrodynamic force (*F*_stokes_) expressed by the Stokes’ equation

(2) $$F_{stokes}=6\pi \eta av$$

where *η* is the surrounding fluid viscosity (Pa s) and ν is the velocity of the flowing cells [m s*^−^*^1^]. If we employ nominal value for the fluid viscosity, η = 1.0 *×* 10*^−^*^3^ (Pa s), the calculated value of the vertical component of *F*_stokes_ to the separator is approximately half compared to that of *F*_DEP_. Thus, RBCs transferred along the separator (i). However, RBCs passed through the gap regions because F_DEP_ for RBCs at the gap regions would decrease below the vertical component of *F*_stokes_. The repulsive force of n-DEP for cells increases with increasing the size of cells in the constant flow rate because the force is in proportion to the cube of cell radius, while hydrodynamic force is proportional to the cell radius. The maximum n-DEP force for THP-1 cells whose size is 2.4 times larger than that of RBCs is also calculated and found to be approximately 180 pN. The force by n-DEP is more than 10 times as large as that of the vertical component of *F*_stokes_. Therefore, THP-1 cells would be guided to the upper side of the main channel without passing through the gap, although the force for n-DEP decreased.

Thus, the cells with different sizes and DEP properties could be separated at the gap effect. The large cells passed through the gaps on the separator (i) will have the opportunity to guide toward the edge by the separator (iii).

### 3.3. Manipulation of RBCs and Human Acute Monocytic Leukemia Cell Line (THP-1 Cells) by n-DEP

**Figure 3:**
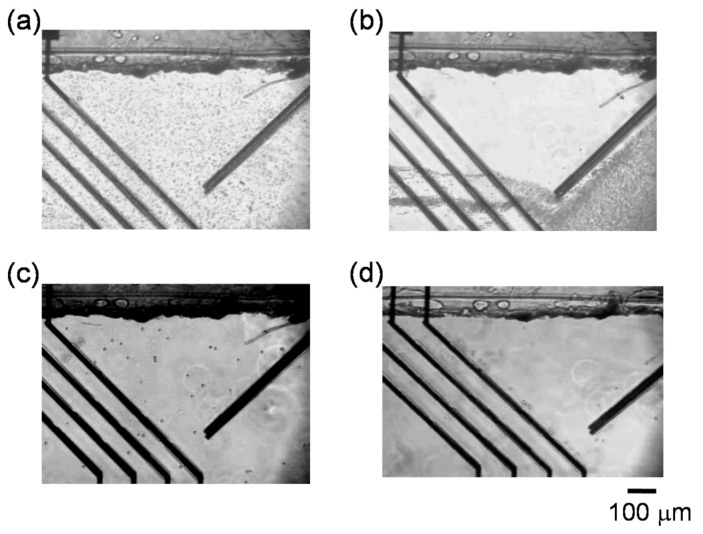
Red blood cells (RBCs) flowing in the channel under negative dielectrophoresis (n-DEP) (**a**) before and (**b**) after applying AC voltage to both navigate electrodes and separate electrodes (i) and (iii). Human acute monocytic leukemia cell line (THP-1 cells) flowing in the channel (**c**) before and (**d**) after applying AC voltage.

Dielectrophoretic properties of RBCs were investigated in the channels to control the fluidic stream of cells. Figure 3a,b shows the regulation of RBCs flowing in the channel under n-DEP (25 kHz). When both navigator and separator electrodes were switched off, RBCs randomly flowed in the channel (Figure 3a). All RBCs flowed along the parallel navigator electrodes after applying AC voltage (30 V_pp_) to the navigator electrodes and thereby accumulation of the suspended cells to the gate arranged at the lower region in the channel (Figure 1c and Figure 3b). Cells flowing in the channel received the repulsive force with n-DEP generated by the navigator electrodes, and flowed along the navigator electrodes after hydrodynamic force was balanced with n-DEP force. Then, AC voltage (14 V_pp_) was applied for separator electrodes (i) and (iii). When the cells accumulated to the gate by the navigator electrode reached at separator electrodes, cells also flowed along the slanted separator electrodes due to the repulsive force of n-DEP. Almost RBCs reached at the gaps prepared on the separator (i) and (iii) flowed to the downstream through the gaps (Figure 3b).

Figure 3c,d show the images of THP-1 in the channel. Although the cells also flowed randomly before applying AC voltage to the navigator and separator electrodes, the cells were accumulated at the gate after AC voltage was applied to the navigator electrodes. When AC voltage (16 V_pp_) was applied to the separator electrodes (i) and (iii), all THP-1 cells directed to the upper side of the channel without passing through the gaps and arrived at the upper edge of main channel (Figure 3d).

**Figure 4:**
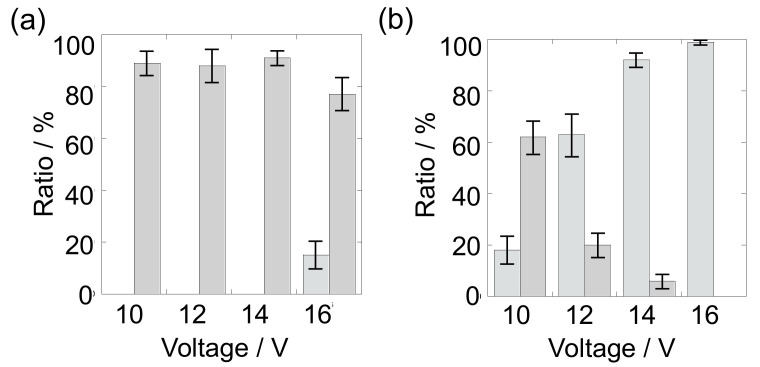
(**a**) Ratio of RBCs reached at upper edge in the channel (bar colored with light gray) and passed through the gaps on separator electrodes (i) (bar colored with gray); (**b**) ratio of THP-1 cells reached at upper edge in the channel (bar colored with light gray) and passed through the gaps on separator electrodes (i) (bar colored with gray).

We investigated the dependence of the applied voltage to the passage efficiency for RBCs and arrival efficiency for THP-1 cells. Figure 4a shows the ratio of RBCs reached at upper edge in the channel (bar colored with light gray) and existed in the main channel after cells were passed through the gaps on separator (i) and (iii) (bar colored with gray). When AC voltages of 10–14 V_pp_ were applied to the separator, RBCs of approximately 90% were passed through the gaps and no RBCs was arrived at upper edge in the channel. The n-DEP force near the gap region is relatively low compared to that generated between the separator. Thus, RBCs would pass through the gaps owing to the insufficient repulsive force to transport RBCs along the separator. Unfortunately, some RBCs were passed through the region except gaps. In the application of AC voltage of 16 V_pp_ to the separator, the ratio of RBCs passed through the gaps decreased slightly, while RBCs arrived at upper edge appeared. The passage of RBCs through the gaps would be hindered because n-DEP force increased with increasing the applied voltage. Figure 4b shows the ratio for THP-1 cells. The application of the AC voltage at 10 V_pp_ was insufficient to bring cells at the upper edge, resulting in the cells over half are passed through the gaps. The arrival efficiency to the upper edge for THP-1 cells increased with increasing the applied voltage, while a ratio passed through the gaps decreased. Almost all cells were arrived at upper edge by 16 V_pp_. The ratio of THP-1 cells arrived at the upper edge is higher than that of RBCs. The n-DEP force, that is proportional to the cube of radius, for THP-1 cells is approximately 8 time larger than that for RBCs, because the average radius of THP-1 cells (7.5 μm) is approximately twice as large as that of RBCs (4.0 μm). Therefore, these results indicate that the intensity of 14 V_pp_ is the most suitable to separate RBCs and THP-1 cells in this experimental condition with the conductivity of 51 mS m*^−^*^1^ in the present device. In this voltage, the passage efficiency for RBCs through the gaps and the arrival efficiency for THP-1 cells to the upper edge in channel were estimated and found to be 91% and 93%, respectively.

We previously reported the capture of cells in microwells with p-DEP [36,37] and the formation of cell aggregates by gathering cells with n-DEP [5]. Cells maintained the esterase activity and proliferation after cells were trapped in microwells to be exposed to the relatively strong electric field. Moreover, cells also maintained their viability even after cells were applied to the electric field generated by an AC voltage of 20 Vpp for 45 min to obtain the cell aggregations. In contrast, cells were only exposed to a relatively weak electric field by the repulsive force against the electric field for approximately 15 s. Thus, the cells used in the experiments would maintain the viability.

### 3.4. Separation of THP-1 Cells and RBCs from Mixtures

**Figure 5:**
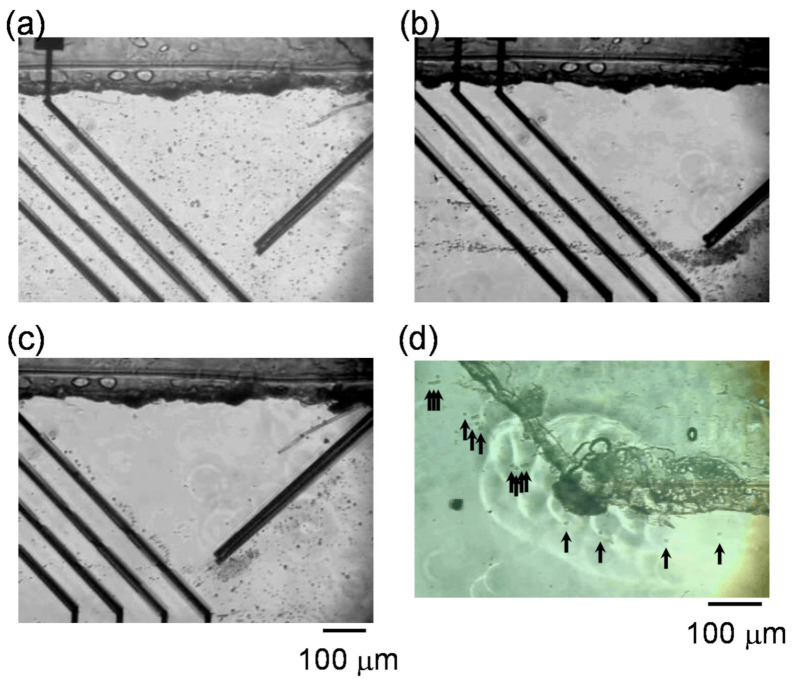
Images of RBCs and THP-1 cells flowing in the channel (**a**) before and (**b**,**c**) after applying AC voltage to both navigate electrodes and separate electrodes (i) and (iii). (**d**) Image of THP-1 cells guided from the main channel to the side channel.

The mixture of RBCs and THP-1 cells was separated by the present microfluidic separator based on n-DEP. AC voltages of 30 and 14 V_pp_ were applied to the navigator and separator electrodes, respectively. Figure 5 shows the series of images of RBCs and THP-1 cells flowing in the channel. Again, before applying AC voltage to the electrodes, both cells parallelly flowed along the channel (Figure 5a). After applying AC voltage, both cells were navigated and accumulated to the gate at the lower edge of the channel with n-DEP and passed through it. When both cells were reached at the separator electrodes (i), they experienced repulsive force of n-DEP to direct toward the upper edge in the channel along the slanted electrodes. At the gap regions, RBCs passed through the gaps to form two lines in the downstream beyond the gaps (Figure 5b). On the other hand, THP-1 cells flowed toward the upper edge along the separator (i) without passing through the gaps. Slight numbers of THP-1 cells that passed through the gaps on the separator (i) flowed toward the upper edge along the separator (iii). Figure 5d shows THP-1 cells guided from the main channel to the side channel. THP-1 cells reached at the upper edge by the separator (i) and (iii) flowed down the main channel along to the upper edge. When THP-1 cells reached at the entrance of the side channel, all cells were guided to the side channel due to the separation of the fluid in the main channel. In contrast, RBCs separated at lower half of the main channel flower down the main channel. Thus, RBCs and THP-1 cells were directed to the different outlets. The result indicates that the slanted electrodes with the different electric field strength by preparing the gaps allows to separating the cells with the different sizes. In addition, no RECs were observed at the side channel in the present experimental condition. The generation of multiple regions with different electric field strengths by establishing the gaps with different widths on the single slanted electrodes (separators) would lead to the multiple separations with their sizes in the multiple fragments.

## 4. Conclusions

In this work, we demonstrate label-free separation of RBCs and WBCs based on n-DEP with an alteration of the electric field distribution generated by slanted electrodes in the microfluidic channels. 3D slanted electrodes with same design on both top and bottom substrates were used as navigator and separator electrodes to deflect the flow of cells laterally. Gaps covered with insulation layer on the separators were prepared to form the alteration of the electric field. Cells reached at slanted electrodes (navigator) were laterally deflected by n-DEP and accumulated to an edge of a channel. Accumulated cells then flowed toward another edge along slanted electrodes (separator) that were placed with an opposite angle. RBCs passed through the gaps on separators, while WBCs were guided to the edge in the channel without passing through the gaps, because of an insufficient repulsive force of n-DEP to RBCs with relatively small size. Therefore, the use of the present system allows to separating cells continuously in a label-free with their sizes. The preparation of different sizes of gaps on separators could lead to separate cells to the multiple sizes in each fragment.

## Supplementary Materials

The following are available online at https://www.mdpi.com/2072-666X/11/9/833/s1, Figure S1: Images of RBCs accumulated by DEP.
