# Supplementary material for: Microfluidic Separation of Blood Cells Based on the Negative Dielectrophoresis Operated by Three Dimensional Microband Electrodes
Source: Micromachines (Basel). 2020 Aug 31;11(9):833. doi: 10.3390/mi11090833 (PMC7570220; doi:10.3390/mi11090833)
Supplement: Supplementary file 1 [file micromachines-11-00833-s001.pdf]

Supplementary Material

Microfluidic Separation of Blood Cells Based on the Negative Dielectrophoresis Operated by Three Dimensional Microband Electrodes

Tomoyuki Yasukawa Jyunko Yamada, Hitoshi Shiku, Tomokazu Matsue and Masato Suzuki

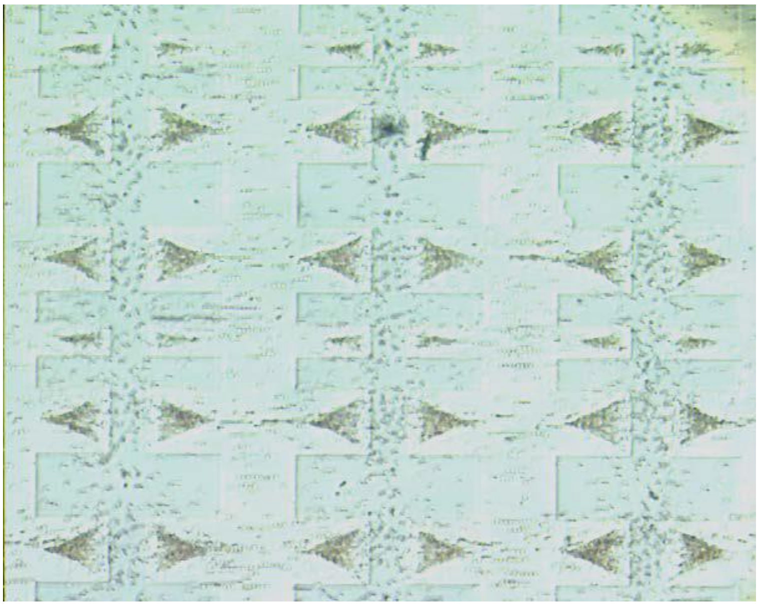

(A)

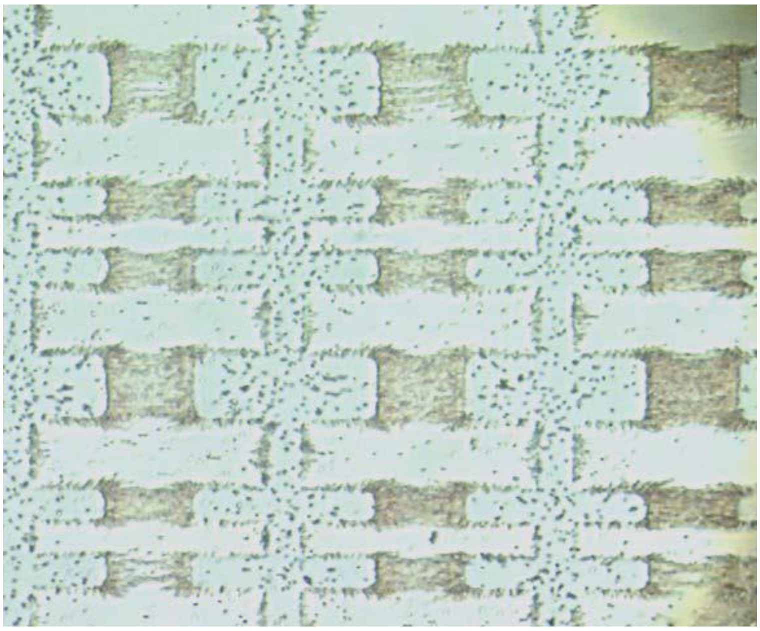

(B)

100 μm

Figure S1. (A) RBCs accumulated on the regions between the projecting parts in the single bands by n-DEP, and (B) RBCs accumulated on the regions between the projecting parts of neighbor bands by p-DEP.

© 2020 by the authors. Submitted for possible open access publication under the terms and conditions of the Creative Commons Attribution (CC BY) license (http://creativecommons.org/licenses/by/4.0/).
